# Laparoscopic treatment of isolated superficial peritoneal endometriosis for managing chronic pelvic pain in women: study protocol for a randomised controlled feasibility trial (ESPriT1)

**DOI:** 10.1186/s40814-020-00740-9

**Published:** 2021-01-07

**Authors:** Lucy H. R. Whitaker, Ann Doust, Jacqueline Stephen, John Norrie, Kevin Cooper, Jane Daniels, Lone Hummelshoj, Emma Cox, Laura Beatty, Patrick Chien, Mayank Madhra, Katy Vincent, Andrew W. Horne

**Affiliations:** 1 MRC Centre for Reproductive Health, University of Edinburgh, Edinburgh, EH16 4TJ UK; 2 Usher Institute, Edinburgh Clinical Trials Unit, University of Edinburgh NINE Edinburgh BioQuarter, Edinburgh, EH16 4UX UK; 3 NHS Grampian, Aberdeen Royal Infirmary, Foresterhill, Aberdeen, AB25 2ZN UK; 4 Nottingham Clinical Trials Unit, University of Nottingham, School of Medicine, Nottingham Health Sciences Partners, Queens Medical Centre, Nottingham, NG7 2UH UK; 5 Endometriosis.org, London, UK; 6 Endometriosis UK, London, UK; 7 NHS Greater Glasgow and Clyde, Queen Elizabeth University Hospital, Glasgow, G51 4TF UK; 8 Ninewells Hospital, Dundee, DD1 9SY UK; 9 NHS Lothian, Royal Infirmary of Edinburgh, Edinburgh, EH16 4SB UK; 10 Nuffield Department of Women’s and Reproductive Health, University of Oxford, Oxford, OX3 9DU UK

**Keywords:** Chronic pelvic pain, Surgery, Excision, Ablation, Feasibility trial, Placebo

## Abstract

**Background:**

Endometriosis (where endometrial-like tissue is found outside the uterus) affects ~ 176 million women worldwide and can lead to debilitating pelvic pain. Three subtypes of endometriosis exist, with ~ 80% of women having superficial peritoneal endometriosis (SPE). Endometriosis is diagnosed by laparoscopy and, if SPE is found, gynaecologists usually remove it surgically. However, many women get limited pain relief from surgical removal of SPE. We plan to undertake a future large trial where women who have only SPE found at initial laparoscopy are randomly allocated to have surgical removal (excision or ablation) of SPE, or not. Ultimately, we want to determine whether surgical removal improves overall symptoms and quality of life, or whether surgery is of no benefit, exacerbates symptoms, or even causes harm. The primary objective of this feasibility study is to determine what proportion of women with suspected SPE undergoing diagnostic laparoscopy will agree to randomisation. The secondary objectives are to determine if there are differences in key prognostic parameters between eligible women that agree to be randomised and those that decline; how many women having laparoscopy for investigation of chronic pelvic pain are eligible for the trial; the range of treatment effects and variability in outcomes and the most acceptable methods of recruitment, randomisation and assessment tools.

**Methods:**

We will recruit up to 90 women with suspected SPE undergoing diagnostic laparoscopy over a 9-month recruitment period in four Scottish hospitals and randomise them 1:1 to either diagnostic laparoscopy alone (with a sham port to achieve blinding of the allocation) or surgical removal of endometriosis. Baseline characteristics, e.g. age, index of social deprivation, ethnicity, and intensity/duration of pain will be collected. Participants will be followed up by online questionnaires assessing pain, physical and emotional function at baseline, 3 months, 6 months and 12 months.

**Discussion:**

Recruitment to a randomised controlled trial to assess the effectiveness of surgery for endometriosis may be challenging because of preconceived ideas about treatment success amongst patients and clinicians. We have designed this study to assess feasibility of recruitment and to inform the design of our future definitive trial.

**Trial registration:**

ClincicalTrials.gov, NCT04081532

**Status:**

Recruiting

## Background

Endometriosis is a chronic oestrogen-dependent condition that affects an estimated 176 million women worldwide [1]. It is defined by the presence of endometrial-like tissue (‘lesions’) outside the uterus [2]. It is now generally accepted that there are three endometriosis subtypes (‘superficial peritoneal’ or ‘SPE’, ‘ovarian’, and ‘deep’). Endometriosis is associated with debilitating pelvic pain and/or infertility and the socioeconomic costs of endometriosis in the UK are ~ £8.2 billion per year with direct healthcare costs amounting to ~ £2315 per woman per year (based on 2009 prices), similar to those of diabetes mellitus [3].

Management options in current national and international endometriosis guidelines for women with endometriosis-associated pelvic pain include surgical removal of endometriosis and medical treatment with analgesics, ovarian suppressive drugs, and neuromodulators [4, 5]. ‘Surgical removal’ involves laparoscopic excision and/or ablation of the endometriosis, often undertaken at the time of initial laparoscopy to investigate pelvic pain. Establishing whether treating isolated SPE in women with chronic pelvic pain is clinically and cost-effective is important because this forms a large part of the workload in gynaecology and uses considerable resources. Around 30% of the direct health care costs of endometriosis are attributable to the cost of surgical treatment. Data from Scotland (population: 5.3 million, 51% women) indicate that 101,137 pelvic laparoscopies were performed in women from 1981 to 2010 [6]. An estimated 91,023 (90%) of these procedures were for investigation of chronic pelvic pain and, of these, 17,834 (20%) revealed a new diagnosis of endometriosis. Half of the women with endometriosis in this population underwent a further surgical procedure for this condition within 5 years.

There is little scientific evidence to demonstrate whether surgical removal of isolated SPE (accounting for ~ 80% of the subtypes) improves overall symptoms and quality of life more than not surgically treating the endometriosis, or whether surgery could exacerbate symptoms (or even cause harm). In the most recent Cochrane review of ‘laparoscopic surgery for endometriosis’, the authors conclude that laparoscopic removal improves ‘condition-associated pain’ (cited as ‘better’ or improved’) compared to diagnostic laparoscopy alone at 6 months (OR 6.58, 95% CI 3.31 to 13.10) [7]. Yet, this conclusion is based on data from only three randomised controlled trials (RCT), only one of which blinded the participants to their allocation, with a total of just 171 participants as well as an amalgam of different subtypes of endometriosis. Furthermore, only one unblinded RCT included in the analysis (just 69 participants) has follow-up data to 12 months showing benefit of surgery (OR 10.00, 95% CI 3.21 to 31.17), leading the authors to define the strength of the evidence as of moderate and low quality, respectively, for the two timepoints, using GRADE criteria [8]. Furthermore, the uncertainty around surgical management of SPE is compounded by the limited evidence to allow an informed selection of specific surgical modalities to remove SPE, e.g. laparoscopic ‘ablation’ versus laparoscopic ‘excision’) [9].

Consequently, the 2017 NICE Endometriosis Guideline recommends further research into the effectiveness of laparoscopic removal of SPE in isolation to manage endometriosis-associated pain [10]. This research recommendation is also supported by the results of a recent UK and Ireland James Lind Alliance Priority Setting Partnership (PSP) Initiative for Endometriosis established to identify the key research questions that were most important to both women with endometriosis and healthcare practitioners involved in their care [9].

If future research demonstrates that surgery is not effective for the treatment of pain associated with isolated superficial peritoneal disease, it is possible that this group of women could be spared an invasive surgical procedure, in particular if their pelvic imaging does not reveal any pathology (pelvic ultrasound or MRI, interpreted by an experienced operator, will diagnose ovarian and deep endometriosis subtypes). They could instead be offered investigations for other known causes of pelvic pain and early pain management (e.g. neuromodulator drugs, physiotherapy, and psychological approaches). Furthermore, it is conceivable that future research might demonstrate that surgery for SPE in isolation is not only ineffective, but may aggravate the symptoms of pain, or even cause harm. It is therefore crucial that policymakers, funding bodies, researchers, clinicians, and women with endometriosis work together in a ‘precision medicine ecosystem’ to build a knowledge base that can determine whether SPE is better suited to surgical, conservative, or multimodal treatment to ultimately guide and improve individualised patient care.

We believe that a large multicentre high-quality RCT is urgently needed to determine whether laparoscopic excision/ablation is of clinical benefit to women with chronic pelvic pain where the only finding is SPE. The aim of this proposed blinded randomised study is to inform the feasibility and design of this future large multicentre RCT.

### Primary objective

The primary objective is to determine what proportion of women with suspected SPE undergoing diagnostic laparoscopy will agree to randomisation to either surgical removal of SPE or diagnostic laparoscopy alone.

### Secondary objectives

1. To determine if there are any differences in key prognostic parameters (including age, social deprivation, ethnicity, and duration of pain) between eligible women that agree to be randomised and those that decline participation.
2. To determine how many women having laparoscopy for investigation of chronic pelvic pain are eligible for the trial (i.e. have only SPE).
3. To determine the variability in outcomes.
4. To determine the most acceptable methods of recruitment, randomisation, and assessment tools.

## Methods/design

### Study design

**Fig. 1:**
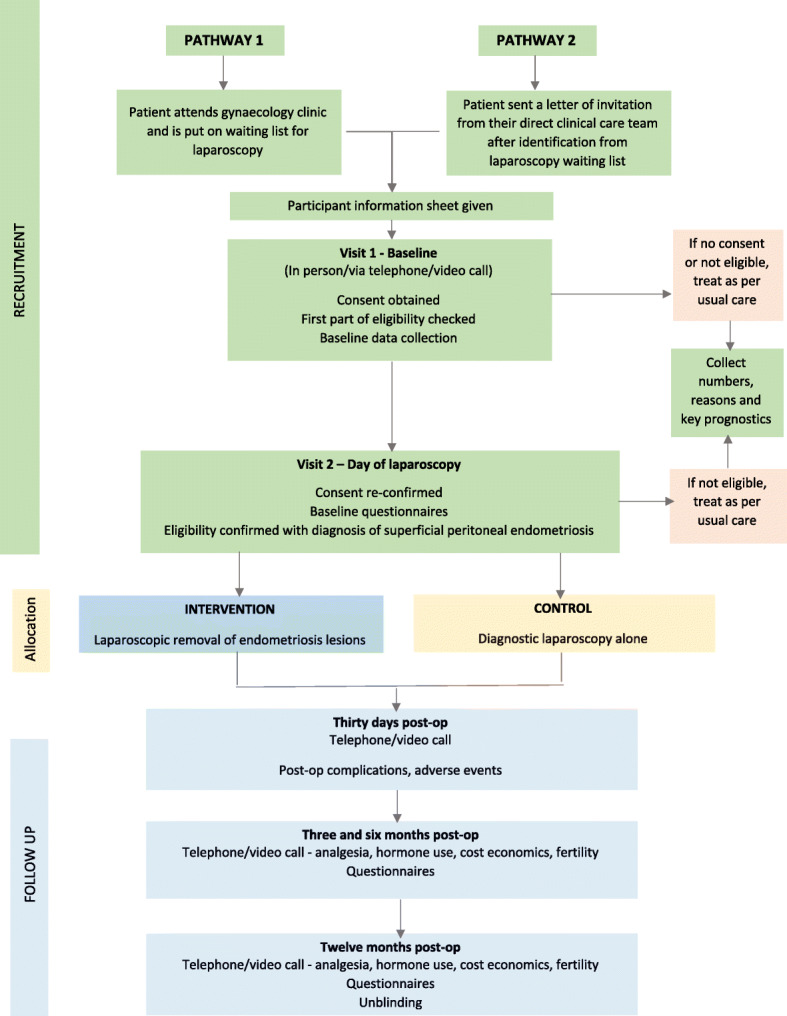
Flow chart for the trial process

We aim to perform a two-arm, parallel, double-blind randomised controlled feasibility trial (Fig. 1).

### Study settings

We will recruit patients from gynaecology outpatient clinics, gynaecology wards, and day surgery units within NHS Lothian (Royal Infirmary of Edinburgh and St John’s Hospital), NHS Grampian (Aberdeen Royal Infirmary), NHS Tayside (Ninewells Hospital) and NHS Greater Glasgow and Clyde (Queen Elizabeth University Hospital, Victoria Infirmary, Glasgow Royal Infirmary and Stobhill Hospital) (all UK).

### Study participants

Women with suspected SPE will be recruited prior to investigation by diagnostic laparoscopy.

### Inclusion criteria

Women are eligible for inclusion if they are as follows:

- Aged between 18 and 55
- Undergoing laparoscopy for the investigation of chronic pelvic pain
- Found to have isolated SPE identified at laparoscopy (macroscopically)
- Able to give informed consent

### Exclusion criteria

Women are excluded if they have one or more of the following:

- Previous surgical diagnosis of endometriosis
- Pregnant
- Deep endometriosis or ovarian endometrioma on imaging or at laparoscopy
- Ovarian cyst requiring surgical management
- Peritoneal ‘pockets’ only noted at laparoscopy

### Interventions

Participants will be randomised to diagnostic laparoscopy alone or surgical removal of SPE. The operating surgeon can choose to remove the endometriosis either by excision, ablation or a combination of the two at their own discretion. All surgeons will be asked to insert two 5 mm lateral wall ports in addition to the 10 mm umbilical port. One lateral wall 5 mm port is always required for a diagnostic laparoscopy. A second lateral wall port is often required to remove endometriosis lesions. Two 5 mm ports will therefore be inserted in order to maintain patient blinding. All participants consented to the study will be informed of other options for the management of endometriosis-associated pain available to them throughout the trial, as part of routine clinical care, including analgesics, ovarian suppressive drugs, neuromodulators and physical and psychological therapy.

### Primary outcome

The proportion of eligible women, who agree to randomisation. If the upper limit of the 95% confidence interval of the proportion of eligible women who agree to randomisation is not less than 40% then this will be deemed an acceptable recruitment rate to proceed to a definitive trial.

### Secondary outcomes

- The reasons why women decline participation
- The proportion of women undergoing diagnostic laparoscopy who go on to be diagnosed with SPE
- Intraoperative and postoperative complications
- Pelvic pain and quality of life
- Need for analgesia, hormonal treatment, and/or neuromodulators after laparoscopy (immediate post-operative period and longer term)
- The proportion of completed trial questionnaires
- Maintenance of blinding
- Adverse events (as reported by the participants)
- Acceptability of the trial

### Participant enrolment

Participants will be recruited from gynaecology outpatient departments following a clinical decision to perform a diagnostic laparoscopy for the investigation of chronic pelvic pain. Potentially eligible women will be asked by their attending clinician if they are happy to meet with a member of the clinical research team. If they agree, the women will then be given a patient information sheet and the opportunity to discuss the trial. A letter of invitation may also be sent to potential participants who have been referred to the research team from the women’s direct clinical care team. Due to the COVID-19 pandemic, initial contact by the research team was widened to include contact by telephone. Consent will only be taken later once the patient has had ample time, of at least 24 h, to read the patient information sheet. A further appointment will be arranged for the participant to come for this visit. Those patients initially contacted by telephone have the option to attend the hospital to discuss the trial and provide written informed consent or provide informed consent verbally over the telephone. All participants will be asked to re-confirm consent on the day of laparoscopy and will be asked to sign the study consent form before any research activities are carried out.

For those not interested in the trial, we will ask them to sign a separate short consent form agreeing for us to keep their age, ethnicity, part of their postcode (to calculate DEPCAT score), duration of pain, and reason for not participating so that we can look at the demographics of those participants who decline, which will help inform a future larger trial. This short consent may take place in less than 24 h as this may take place in a clinic setting and will reduce the burden of further contact on the participant. If eligible participants were not approached, we will ask the attending clinician to document their reasoning for this. All data will be recorded on a case report form and transferred to a secure database. The diagnosis at laparoscopy will be established for all eligible women for the duration of the trial to establish the true incidence of SPE in isolation.

### Sample size

The emphasis in this study is to establish feasibility, not statistical significance. This study is designed primarily to explore recruitment rates, and we will aim to recruit as many women as possible over a 9-month period. We estimate that we will recruit ~ 2–3 women per month per centre and will aim to recruit up to 90 women. Ninety participants would be able to give a half width of the 95% confidence interval of +/− 6.4% around a 40% recruitment rate.

### Randomisation

Eligibility will be confirmed by the clinical research team at the time of laparoscopy following a finding of SPE only. Participants will then be randomised by the clinical research team in a 1:1 ratio to either diagnostic laparoscopy alone or to concurrent surgical removal (ablation/excision) by simple randomisation using a computer-generated random number list provided independently by the Edinburgh Clinical Trials Unit (ECTU). As randomisation takes place intra-operatively, it will be a fast, simple procedure in theatre via a web-based system, or via a telephone call to the trial management team who will use the computer system on their behalf.

### Blinding

At the time of consent, the participant will be reminded that they will be given a diagnosis post-operatively of the findings at the time of laparoscopy but will not be told if surgical removal of endometriosis was carried out. They will be told that the details of whether the endometriosis was removed, or not, will not be disclosed to their GP. Details of trial participation and diagnosis will be disclosed to the GP as well as any recommended treatment from their attending physician. There will be a process by which they can be unblinded at their request prior to the formal unblinding process at 12 months. Details of the extent and type of removal (ablation, excision or both) will be recorded. All of the surgical findings and any surgical procedures to remove the endometriosis will be available in the medical notes and there will be information about the participation in the trial and the importance of maintaining the participant blind. At the end of participation, the participants’ GPs will be informed if surgical removal was performed.

### Data collection tools

Our ‘trial questionnaire’ comprises of a range of validated patient-reported questionnaires, collected pre-operatively and then at 3, 6 and 12 months’ post-surgery (minimum data collection timepoints). The baseline and follow-up trial questionnaires will differ in that as follows:

1. The baseline questionnaire contains text prior to the EHP-30 as follows “This questionnaire asks about symptoms due to endometriosis. We realise that you do not know whether or not you have endometriosis so please try and ignore the references to endometriosis and simply answer the questions focusing on the symptoms.”
2. The questions in the MYMOP2 questionnaire have been adapted in the follow-up questionnaires to account for the fact that the participant needs to remember how they answered this questionnaire at baseline.

The ‘trial questionnaire’ will include the following:

- Endometriosis Health Profile-30 (EHP-30) [11]
- Rome IV Criteria [12]
- Pelvic Pain and Urgency/Frequency Patient (PUF) Symptom Scale [13]
- painDETECT™ [14]
- Brief Fatigue Inventory (BFI) [15]
- Pain Catastrophising Questionnaire (PCQ) [16]
- Fibromyalgia Scale (FS) [17]
- Measure Yourself Medical Outcome Profile 2 (MYMOP 2) [18]
- Working Productivity and Activity Impairment Questionnaire (WPAIQ) [19]
- EuroQol 5 Dimensions 5 Level Questionnaire (EQ-5D-5L) [20]
- Capability Questionnaire (ICECAP-A) [21]

At 3, 6 and 12 months post-randomisation, participants will be asked questions (‘outcome questionnaire’) about analgesic use, hormonal medication, pregnancy, and the use of healthcare services. At 3 months post-surgery, participants will also be asked to complete an additional questionnaire (‘acceptability’) that will include questions about the questionnaires and the acceptability of trial participation. This will be via a telephone call with a member of the research team.

### Details of surgical findings and surgical removal of endometriosis

Following laparoscopy, the surgeon will be asked to complete a surgical case report form (SCRF) detailing information from the operation:

- Surgeon’s name(s)
- Extent of endometriosis
- Duration of surgery
- Concurrent intra-uterine system (IUS) insertion
- Intraoperative complications (bowel injury, ureteric injury, bladder injury, vascular injury and reverting to laparotomy)

If allocated to surgical removal of endometriosis:

- Type of removal will be recorded (excision, ablation or both).
- Subjective assessment of whether or not complete removal was achieved.

In addition, the SCRF includes a specific set of pre-determined photographs. For the diagnostic laparoscopy, this is a standard panel of photographs of the pelvis, which includes the utero-vesical fold, Pouch of Douglas and right and left ovarian fossa. If allocated to surgical removal, the approach (ablation, excision or a combination) will be documented; as well an assessment of the adequacy of removal and photographs of the areas after the endometriosis has been removed. Two independent clinicians will assess these images, blind to initial classification, to reduce the likelihood of misclassification.

### Post-operative complications

A post-operative complications form (part of the CRF) will be completed detailing complication up to 30 days post-operation. This will be completed by the clinical research team, utilised information gained by a phone call to the participant and correlated with the participant’s hospital record. Specific complications include urinary retention, unintended overnight stay, haemorrhage, transfusion pelvic haematoma, visceral injury (bowel, bladder, ureteric), infection (urinary, chest, wound, pelvic abscess, other), venous thromboembolism, fistula, hernia, return to theatre, readmission, ITU admission and death.

### Unblinding

An unblinding facility will be available on the database. If a clinician would like to unblind a participant (e.g. at the participant’s request), they will need to contact the research team who will be able to tell them which arm of the study they were in. Participants will be given the option to be unblinded at the end of the trial.

### Adverse events

Participants will be asked about the occurrence of adverse events related to their surgery and related medical therapies, which will be recorded in the post-operative CRF at 30 days.

### Data analysis

Analysis of the quantitative data will largely consist of descriptive statistics and comparison between the two groups. Throughout, we will summarise continuous variables using the mean, standard deviation, median, lower quartile, upper quartile, minimum, and maximum values. We will report categorical variables using the frequency and percentage for each category. Baseline demographic and clinical data will be summarised by study arm and overall. The feasibility outcomes (proportion of eligible women who agree to randomisation, the proportion who decline participation, the proportion who are diagnosed with SPE and the type of surgical removal) will be analysed by calculating the proportion and its exact binomial 95% confidence interval. Clinical and patient outcomes will be summarised by study arm and overall and reported as a difference between study arms. The acceptability of trial participation, proposed methods of recruitment, randomisation, and assessment tools will be assessed quantitatively using the acceptability questionnaire to explore comparisons between the two groups and will be reported as estimates of difference. The variability in the EHP-30 pain domain score and other patient-reported outcome scores will be used to inform the sample size calculation for the full trial. We will qualitatively review the acceptability questionnaire and determine the nature and number of unanswered questions within the clinical questionnaire to refine our assessment tools for the future definitive trial.

### Data handling, storage and archiving

A log with the participants’ name and date of birth will be kept along with their unique study number in a separate file. All the data generated from the study will be stored in a pseudo-anonymised form in a bespoke database, which will also be password protected. Only anonymised information will be stored on this, and participants will only be identifiable by their study number. All paperwork will be kept in a locked filing cabinet in a locked office. All data will be stored on a university server on a password-protected computer with access limited to the research team, in accordance with NHS and University of Edinburgh guidelines, and in accordance with the Data Protection Act. All study documentation will be kept for a minimum of 5 years from the protocol-defined end of study point. When the minimum retention period has elapsed, study documentation will not be destroyed without permission from the Sponsors (NHS Lothian and The University of Edinburgh). The statistical analysis plan will be written prior to unblinding but the final analysis will be done unblinded.

### Data monitoring

No data monitoring committee will be convened for this small feasibility study as all care is standard care or less. The trial management group will ensure that any adverse events are reported in a timely manner to the sponsor for review.

### Trial sponsors

The trial is co-sponsored by the University of Edinburgh and NHS Lothian.

### Ethical approval

Ethical approval has been obtained from the East of Scotland Ethics Committee (NHREC 19/ES/0079).

### Dissemination

Data will be published in peer-reviewed journals and presented at international conferences. The clinical study report will be used for publication and presentation at scientific meetings. We will make the information obtained from the study available to the public through national and international bodies, e.g. Endometriosis UK and Endometriosis.org.

## Discussion

We recognise that there may be difficulties in recruiting to a definitive randomised controlled trial (RCT) to assess the effectiveness of surgery for the painful symptoms associated with superficial peritoneal endometriosis (SPE). We have therefore designed this study to assess the feasibility of recruitment and to generate adequate data to meaningfully reduce uncertainties in other important aspects of the future trial.

A systematic review of surgical RCTs with a placebo arm found that slow recruitment, due to difficulties finding eligible patients (and clinicians) who would agree to participate, was the major barrier to successful trial completion [22]. Patients and clinicians may have inherent beliefs and preferences about surgery as an intervention, which might affect their willingness to participate in a surgical trial with a placebo arm. In this study, we are investigating a surgical procedure that is already widely accepted and available, despite an absence of high-quality evidence supporting its use. We conducted a national online survey of clinicians who manage women with endometriosis in 2017. After excluding those that did not perform laparoscopies, we had 88 responses. When asked to judge the strength of the evidence for ablation and excision for endometriosis, where zero is very weak and 10 very strong, the average weights were 5.9 and 4.3, respectively. Furthermore, 81% of surgeons consider a trial to be required, and 73% are willing to participate. We also conducted an online survey targeting women with endometriosis with promotion from Endometriosis UK, the Endometriosis Association of Ireland, and Endometriosis.org. We received responses from 1218 women, of whom 98.8% had endometriosis, and 83% had their endometriosis treated surgically. When asked whether they would consider taking part in the proposed definitive trial, 20% ‘definitely would’, 26% ‘would likely participate’, and 17% ‘did not know’.

To improve recruitment, we aim to identify and engage with all relevant stakeholders and to develop and test tailored messages and educational materials [23, 24]. We will involve patient representatives and members of the relevant professional surgical societies throughout the study to help increase buy-in of the trial by highlighting the importance of the research question and the worthiness of the placebo design.

A potential limitation (but also a strength) of our study is the broad inclusion and exclusion criteria, which we have produced in an attempt to reflect the true clinical scenario of endometriosis. Our criteria do not take into account pain intensity, do not exclude women with non-reproductive comorbidities (e.g. irritable bowel syndrome, bladder pain syndrome) that could explain their symptoms, and allow participants the use of concomitant medications. We are aware that these characteristics may increase variability in patient responsiveness to treatment and consequently carry the risk of failing to demonstrate treatment effect. We will therefore capture this information in our study in the participants’ questionnaires to ensure informed interpretation of our results as well as aid in the planning of the future definitive trial.

We have chosen data collection tools to assess the core domains of pain, physical/emotional functioning (including sleeping difficulties), improvement/satisfaction with treatment, symptoms and adverse events, following advice from a pain medicine specialist and clinical psychologist. These tools are in line with the IMMPACT recommendations for chronic pain trials [25, 26]. We will assess the reliability and acceptability of these data collection tools in order to refine our methods for the subsequent RCT.

We acknowledge that blinding, whilst essential for an objective reporting of pain by the women, presents additional challenges. In our clinician survey, when surgeons were asked whether they would be prepared to insert an extra port (total of two 5 mm ports in addition to the camera port) at the time of surgery to blind women to treatment allocation, 58% of surgeons agreed. We will monitor the compliance with this extra measure in the diagnostic laparoscopy only group, and survey the acceptability amongst the participants.

In summary, this study with embedded evaluation of trial processes and collection of outcome data, will allow us to undertake detailed feasibility work to inform a future large-scale trial in the important but challenging area of surgery for endometriosis-associated pain.

## Data Availability

Not applicable

## Abbreviations

ECTU: Edinburgh Clinical Trial Unit
REC: Research Ethics Committee
SPE: Superficial peritoneal endometriosis
RCT: Randomised controlled trial
CRF: Case report form
SCRF: Surgical case report form
DEPCAT: Deprivation category
